# Study on South African Indigenous Teas—Antioxidant Potential, Nutritional Content, and Hypoxia-Induced Cyclooxygenase Inhibition on U87 MG Cell Line

**DOI:** 10.3390/molecules27113505

**Published:** 2022-05-30

**Authors:** Motlalepula Gilbert Matsabisa, Asis Bala, Satyajit Tripathy, Michelle Mogomane Digashu, Fanie Rautenbach, Barsha Dassarma, Joseph Omorogiuwa Erhabor, Fernao Castro Braga, Pulok Kumar Mukherjee, Minke Tang, Youngmin Kang

**Affiliations:** 1 Department of Pharmacology, Faculty of Health Sciences, School of Clinical Medicines, University of Free State, Bloemfontein 9300, South Africa; asisbala_ju@yahoo.co.in (A.B.); tripathys@ufs.ac.za (S.T.); digashumm@ufs.ac.za (M.M.D.); dassarmab@ufs.ac.za (B.D.); erhaborjo@ufs.ac.za (J.O.E.); 2 Division of Pharmacology, Guru Nanak Institute of Pharmaceutical Science and Technology, Panihati, Kolkata 700114, India; 3 Faculty of Health and Wellness Sciences, Oxidative Research Institute, Cape Peninsula University of Technology, Cape Town 7530, South Africa; rautenbachf@cput.ac.za; 4 Faculty of Pharmacy, Universidade Federal de Minas Gerais, Belo Horizonte 31270-901, Brazil; fernao@netuno.lcc.ufmg.br; 5 School of Natural Product Studies, Department of Pharmaceutical Technology, Jadavpur University, Kolkata 700032, India; pulokm@yahoo.com; 6 Department of Pharmacology, School of Pharmacy, Beijing University of Chinese Medicines, Beijing 100191, China; tangmk@bucm.edu.cn; 7 Propagation and Production of Traditional Herbal Medicines, Herbal Medicines Resources Centre, Korean Institute of Oriental Medicine, University of Science and Technology, Daejeon 34054, Korea; youngmiin.k@gmail.com

**Keywords:** indigenous tea, commercial tea, antioxidant capacity, nutritional content, cox activity

## Abstract

**Background:** This study comparatively assessed seven indigenous traditional tea plants on several attributes that included antioxidant, nutritional, caffeine contents, and cyclooxygenase activity. **Methodology:** Nutritional content of all tea plants were determined for energy, fat, carbohydrates, total sugars, dietary fiber and amino acids. Antioxidant potential and the antioxidant potentiating secondary metabolites were also measured and compared. Further, we investigated the tea plants for any role they would have on cyclooxygenase (COX) activity on cobalt chloride (CoCl_2_) induced human glioma cell lines (U87MG). **Results:** The tea plants were found non-cytotoxic at concentrations tested against the human Chang liver and HeK 293 kidney cells and were found to be naturally caffeine free. The lowest and highest extraction yield among the tea plants was 7.1% for *B. saligna* and 15.48% for *L. scaberrimma* respectively. On average, the flavonol content was 12 to 8 QE/g, ORAC 800 µmol TE/g, TEAC 150 µmol TE/g, FRAP 155 µmol AAE/g, polyphenols 40 mg GAE/g, flavanols 0.35 mg CE/g, flavonols 12 mg QE/g and total flavonoid content (TFC) 180 µg QE/mg. The COX activity has been found to be inhibited by a dose-dependent manner by *L. scaberrimma*, *B. saligna* and *L. javanica*. **Conclusion:** The results further support competitive value of tea plants and need for improved and further development.

## 1. Introduction

Tea is a popular refreshing drink consumed worldwide. Tea contains polyphenols, which have antioxidant activities and prevent oxidation of low-density lipoprotein (LDL) that may lead to high cholesterol levels [1]. The biological activity of any tea extract is dependent on the presence of the phytochemical constituents, particularly flavonoids, and its processing [2]. 

The traditional uses of indigenous herbal infusions in tropical Africa are recorded in various literatures [3]. The use of these herbal infusions was not only limited to their use as tea but also as health beverages based on their health and medicinal properties [4,5,6]. Indigenous tea plants have been used for centuries because of their nutritional and medicinal properties [7].

Another health benefit that has been linked to tea is its potential against oxidative stress. Oxidative stress has been implicated in diseases such as cancer, diabetes and Alzheimer’s [8]. It leads to the generation of free radicals such reactive oxygen species (ROS), reactive nitrogen species (RNS), superoxide (O_2_^−^), and hydroxyl radicals (OH-). Human cells possess an antioxidant defense system for mopping up free radicals by donating an electron to quench their instability [9]. However, continuous generation of free radicals causes a shift from the oxidant–antioxidant balance in favor of the former, thereby leading to oxidative stress. Antioxidants in plants are good defense mechanisms for scavenging carcinogenic free radicals. In addition, hypoxia is a state in which a tissue’s oxygenation is insufficient. When exposed to hypoxia, tissues/cells can be made to live by raising glycolysis rate [10]. However, hypoxic microenvironments are beneficial to cancer cells, tumor development, invasion, and angiogenesis [11]. The elevation of vascular endothelial growth factor (VEGF) expression caused by hypoxia is an important stage in neovascularization. Hypoxia has been demonstrated to promote angiogenesis and metastasis by stimulating cyclooxygenase (COX), an inducible enzyme that catalyzes the synthesis of prostaglandins (PGs) from arachidonic acid.

**Figure 1:**
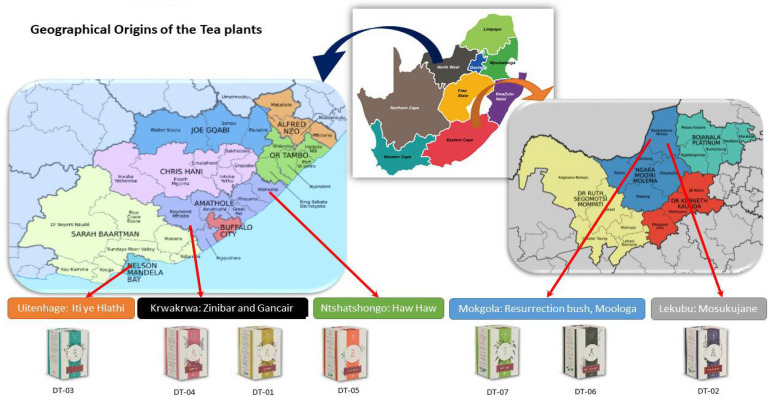
Geographical origins of the Indigenous tea plants-infusions.

According to South African Tea Industry Landscape Report 2016, South African tea consumers have a shifting preference to consuming the indigenous or local tea. Therefore, the economic importance of the indigenous tea plants is growing day by day. So, the aim of this study is to evaluate the in vitro cytotoxicity, nutritional benefits, and effects on COX activity of hypoxia-induced human glioma cell line on identified (through collaborative work with local indigenous communities) seven indigenous South African indigenous tea plants (Supplementary Figure S1). These include *Croton gratissimus* var *gratissimus* (L.). *Myrothamnus flabellifolius* (Welw.). *Lippia scaberrimma* (Sond.), *Lippia javanica* (Burm.f.) Spreng, *Buddleja saligna* (L.), and *Phyla dulcis* (Trevir.). The botanical names, family, common and local names, as well their medicinal uses, pictures cum geographical coordinates/origins are outlined in Table 1 and Figure 1.

## 2. Results 

### 2.1. Percentage (%) Yield of Different Tea Extracts

Extraction yield of tea plants calculated as an extract to raw plant weights ranged from 7.10–15.48% (*w*/*w*) (Table 2). The DT-02 extract had the highest yield of 15.48% (Table 2).

### 2.2. The FRAP Assay

**Figure 2:**
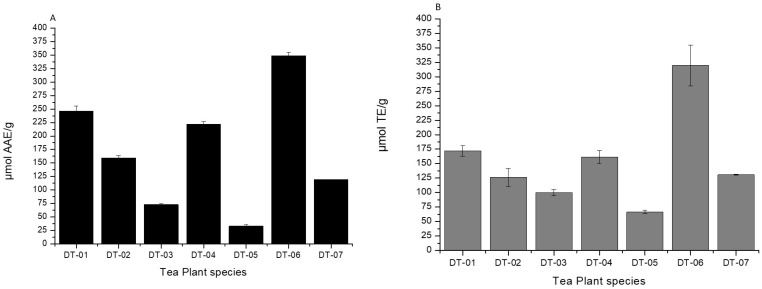

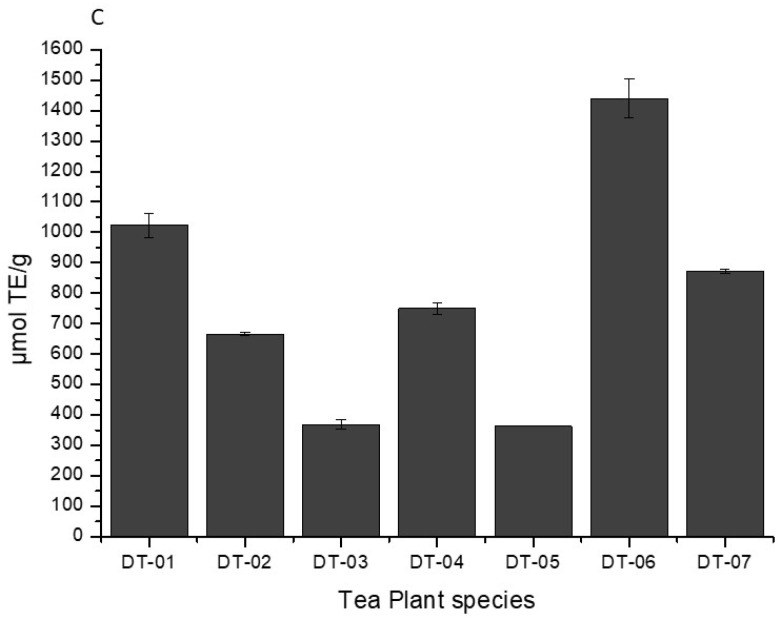
Antioxidant capacities of all the tea plants. (**A**) FRAP; (**B**) TEAC; and (**C**) ORAC levels. *n* = 3.

The highest reducing power capacity was observed for DT-06, and this was more than twice the calculated average value (171.74 ± 4.08 µmol AAE/g) of indigenous tea plants (Figure 2A). However, there was a significant difference found between DT-03 and DT-05 (Figure 2A). In contrast, the indigenous tea DT-06 was found to have maximum FRAP capacity (349.138 µmol AAE/g) (Figure 2A) equivalent to a reducing power of 872.846 µmol AAE/2.5 g, an equivalent amount in one standard tea bag. 

### 2.3. The ABTS Radical Cation-Scavenging Assay: Trolox Equivalent Antioxidant Capacity (TEAC)

In the ABTS radical cation-scavenging assay to estimate Trolox Equivalent Antioxidant Capacity (TEAC) of all tea plants. DT-06 showed higher scavenging capacity (319.36 µm TE/g) than average value (153.74 ± 11.55 µm TE/g) for indigenous tea plants. All other tea plants, except DT-03 and DT-05, which had least average antioxidant capacity, showed equivalent scavenging activity comparable to more than the average value (Figure 2B). The average TEAC for the commercial teas was found to be 180.58 ± 10.68 µm TE/g. However, DT-06 had the most antioxidant capacity.

### 2.4. Trolox Oxygen-Radical Absorbance Capacity (ORAC)

The average ORAC was found to be 783.00 ± 21. 27 µmol Trolox Equivalent/gram (µmol TE/g) of extract for indigenous tea plants. DT-06 showed higher antioxidant capacity (1500 µmol TE/g) than average value. However, DT-03 and DT-05 had the least antioxidant capacity than average value at 400 µmol TE/g each). All other tea plants showed antioxidant capacity above the average value (Figure 2C). However, higher ORAC activities were observed for DT-01 and DT-06, and DT-06 showed maximum ORAC capacity (1500 µmol TE/g) (Figure 2C).

### 2.5. Nutritional Content Estimation

The result of the nutritional content of all the tea plants is presented in Table 3. The tested tea plants had low levels of natural sugars (below 30%) and with less than 1% of water-soluble carbohydrates. All tea plants had high total dietary fiber levels ranging from (30–60)%. All tea plants contained essential amino acids ranging from (0.5 to 2) g/100 g (0.5 to 2)%. 

The energy levels of all tea plants were found to be on average 26–38 KJ/100 g with averages of (33. 3 ± 5.187) KJ/100 g in indigenous tea plants. The total fat value for all tea plants were between 0.71% and 1.06% while the average was (0.9043 ± 0.1412)% for indigenous tea plants (Table 3B). The average moisture content and ash values for all the tea plants were comparable; (99.7471 ± 0.0869) % for indigenous tea plants. Total soluble solids on average were (0.2557 ± 0.0913)% in indigenous tea plants. 

#### 2.5.1. Polyphenols Equivalent to Gallic Acid Estimation

**Figure 3:**
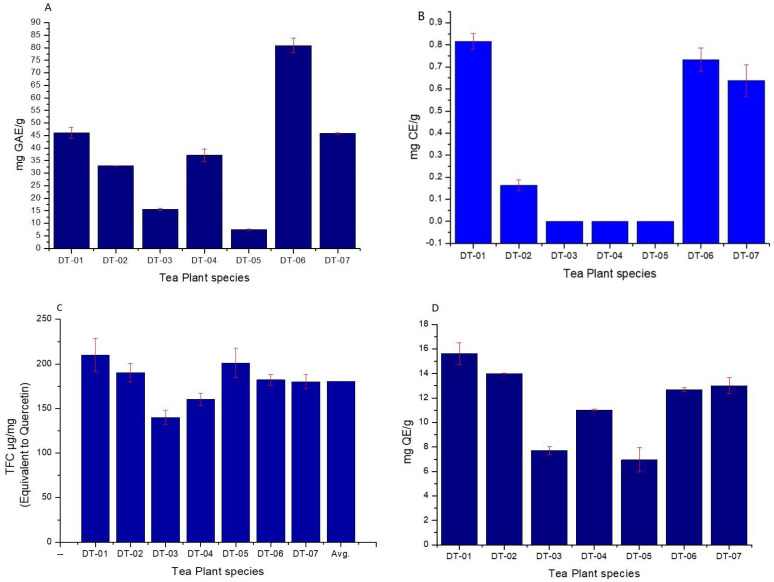
Evaluation of antioxidant compounds in all the tea plants. (**A**) Estimation of polyphenols equivalent to gallic acid; (**B**) estimation of flavanols equivalent to catechin; (**C**) total flavonoid content (TFC) estimation; (**D**) estimation of flavonols equivalent to quercetin.

Approximately, twice higher the amount of average quantity of polyphenols equivalent to gallic acid, was found for DT-06 (349.138 µmol AAE/g). The highest polyphenol equivalent to gallic acid was 80.90 ± 2.85 mg. However, the lowest polyphenols (7.52 ± 0.23 mg) were found in DT-05 and that was less than the average value (Figure 3A). DT-06 (80. 90 mg GAE/g) showed polyphenols levels more than the average value (Figure 3A).

#### 2.5.2. Total Flavanols Estimation as Catechin Equivalent

All indigenous tea plants had negligent to no flavanol content (Figure 3B).

#### 2.5.3. Total Flavonoid Content (TFC)

Average TFC found 193.40 ± 10.51 µg/mg of indigenous tea plants respectively (Figure 3C). Interestingly, for indigenous tea plants, 5 out of 7 (DT-01, DT-02, DT-05, DT-06 and DT-07) tea plants have TFC values above the average value limit. However, none of the samples had an increased average quantity of TFC when compared using the appropriate statistical tool. 

#### 2.5.4. Flavonols Equivalent to Quercetin Estimation

The average value for flavonols was 11.57 ± 0.44 mg QE/g. All tea plants except DT-03 (7.717 mg QE/g) and DT-05 (6.947 mg QE/g) showed less flavonols content levels. DT-01 (15.627 mg QE/g) and DT-02 (14 mg QE/g) had the highest flavonols content. Interestingly, DT-01 (15.627 mg QE/g) showed higher amounts of flavonols as compared to average value (Figure 3D). 

### 2.6. Cytotoxicity and Cell Proliferation

**Figure 4:**
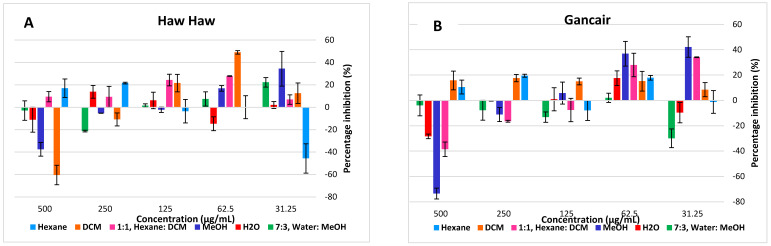

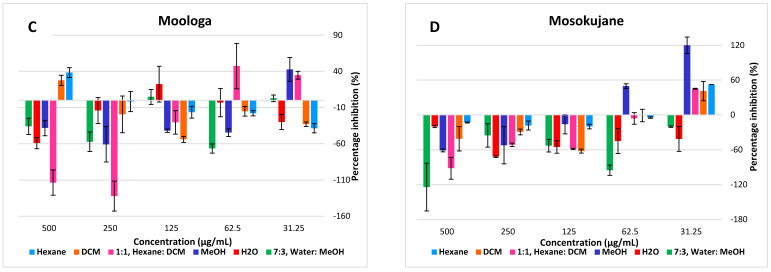

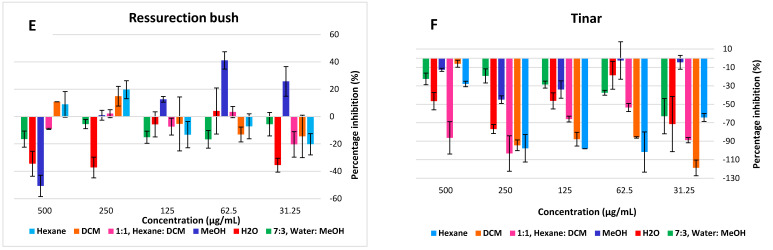

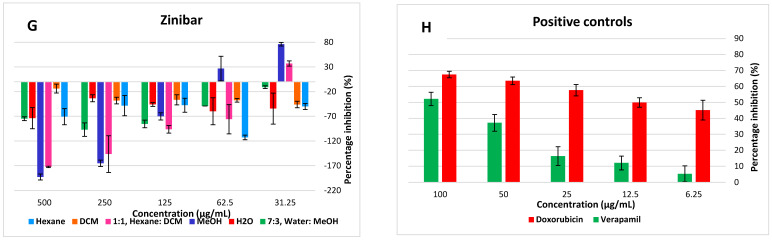
Cytotoxicity and cell proliferation on normal human Chang Liver cells over 24 h exposure of tested indigenous tea plants with trade names ((**A**) Haw; (**B**) Gancair; (**C**) Moologa; (**D**) Mosukujane; (**E**) Resurrection bush; (**F**) Tinar; (**G**) Zinibar. (**H**) Positive controls).

**Figure 5:**
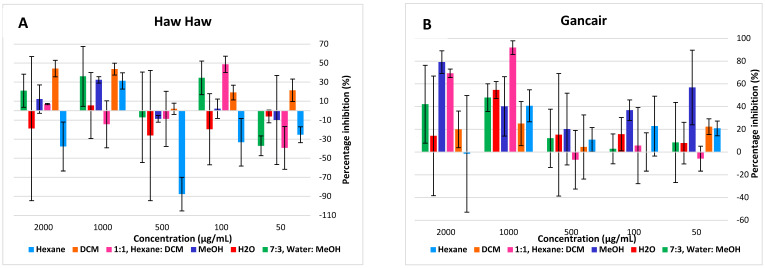

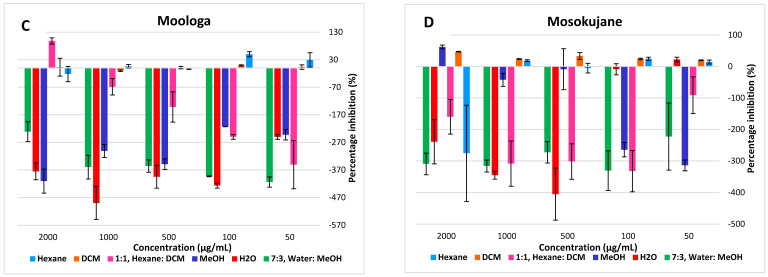

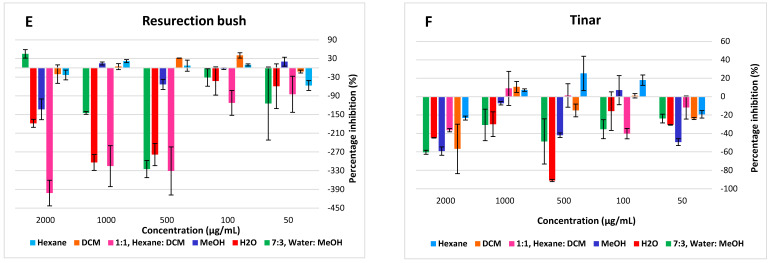

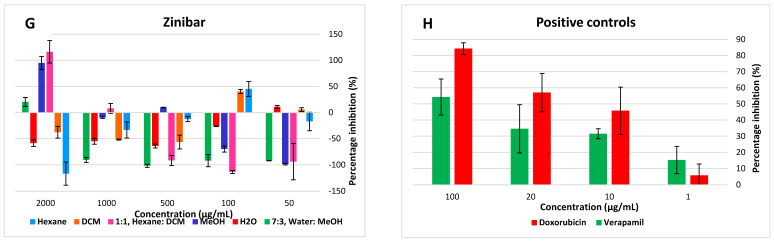
Cytotoxicity and cell proliferation on normal human HEK293 liver cells over 24 h exposure of tested indigenous tea plants with trade names ((**A**) Haw Haw; (**B**) Gancair; (**C**) Moologa; (**D**) Mosukujane; (**E**) Resurrection bush; (**F**) Tinar; (**G**) Zinibar. (**H**) Positive controls).

The water extracts of the tea plants were tested at different concentrations over 24 h exposure, and had no cytotoxic effect on both cell lines but rather showed stimulated cell proliferation (Figure 4 and Figure 5). The cytotoxicity tested on human Chang liver showed none of the infusions had a cytotoxic effect even at the highest concentrations of 500 µg/mL (Figure 4A–H). Similarly, the infusions were not cytotoxic to human HeK 293 kidney cells. DT-04 and DT-05 organic extracts, however, showed some cytotoxicity to kidney cells (Figure 5A–H).

#### Cell Proliferation and COX Inhibition

**Figure 6:**
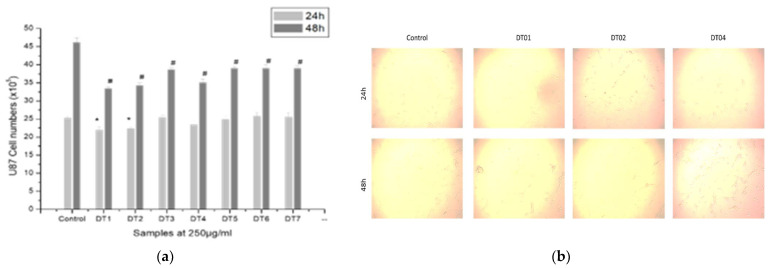
(**a**) Graphical presentation represents the u87 MG cell number after treatment with tea plants extracts (DT01–DT07) at 250 µg/mL for 24 h and 48 h. Values are expressed as mean ± SEM, *n* = 3. * Significant difference (*p* < 0.05) compared to 24 h control group. # Significant difference (*p* < 0.05) compared to 48 h control group. (**b**) Light microscopic images of cell proliferation of Control, DT01, DT02, and DT04-treated groups at different times.

**Figure 7:**
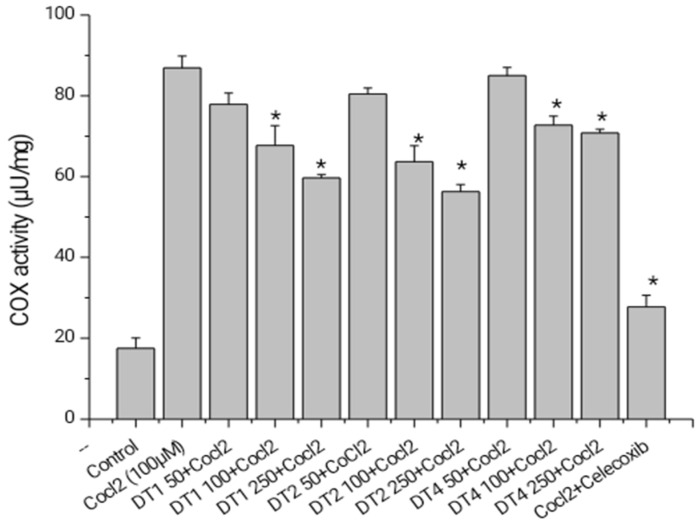
Graphical presentation represents COX inhibition activity in CoCl_2_ (100µM) exposed U87 MG cells after treatment with tea plants extracts (DT01, DT02, and DT04) at 50, 100, and 250 µg/mL for 24 h. Here, celecoxib was used at 100 µM. Values are expressed as mean ± SEM, *n* = 3. * Significant difference (*p* < 0.05) compared to only CoCl_2_ group.

The results (Figure 6) show that tea has a role in the inhibition of U87 MG cell proliferation. The results of the effect of the seven indigenous tea plants extract on cell proliferation of human glioma cell lines were further analyzed. We selected three extracts (DT01, DT02, and DT04) for studying the COX inhibitory activity based on the nutritional contents, cytotoxicity, and by comparing the cell count study. Figure 7 shows that the extracts have COX inhibitory role on CoCl_2_-stimulated U87 MG cells in a dose-dependent manner. CoCl_2_ is commonly used as a hypoxia-mimetic agent. So, in the in vitro glioma cell culture, to generate hypoxic condition, to check the effect of our tea extracts, we exposed the cells in CoCl_2_.

## 3. Discussion

Generally, tea contains polyphenols, alkaloids, amino acids, proteins, volatile compounds, minerals, and trace elements and many other phyto-pharmaceuticals [30]. Previous reports adduce to the fact that tea may confer some health benefits [1]. The essential oils in tea leaves are well-known for their antioxidant properties [31]. In the present study, all the seven indigenous tea plants were examined for their nutritional content with respect to energy, fat, carbohydrates, total sugars, dietary fiber, and amino acids content. Antioxidant potential was measured by FRAP, TEAC, and ORAC assay methods and the antioxidant potentiating secondary metabolites such as polyphenols (mg GAE/g), flavanols (mg CE/g), flavonols (mg QE/g), and total flavonoid content (TFC) (µg QE/mg) were also measured and compared. 

The indigenous tea plants displayed excellent radical scavenging activity in the various antioxidant assays performed. Interestingly in this study, the indigenous tea, DT-06, had almost double the value as compared to average for the best antioxidant capacity based on the FRAP, TEAC, and ORAC when compared to the other tea plants. The antioxidant activity of the tea plants may probably be attributable to the presence of the high quantities of polyphenols and flavonols found in these indigenous tea plants. The highest polyphenols equivalent to gallic acid was found in the DT-06 sample at 349.138 µmol AAE/g. However, for total flavanols estimation as catechin equivalent was found negligent to no flavanol content (<1 mg CE/g). TFC and flavonols equivalent to quercetin estimation content for DT-06 were found to be above the average value of the indigenous tea plants tested (Figure 3A–D). 

Caffeine is the most widely consumed psychoactive drug, and one of the comprehensively studied chemical [32]. The caffeine content of the selected indigenous tea plants was determined, as most consumers now make a preference for caffeine-free beverages, because of its potential harm, especially in babies [33,34,35]. Caffeine at concentrations of more than 400 mg per day may cause insomnia, nervousness and restlessness, stomach irritation, nausea and vomiting, increased heart rate and respiration, and other associated side effects [33,34]. Therefore, consumer consciousness toward caffeine-free health drinks is increasing [35]. Importantly, in the present study, there was no caffeine found in all the assessed tea plants. 

The health benefits of tea are not only associated with antioxidant activities but also include their nutritional and nutraceutical properties [36]. In this study, the energy calculations were performed using total solids, total moisture, and total Ash value estimation, (Average = 33.2857 ± 5.2857 KJ/100 g) (Table 3B). The indigenous tea plants had energy levels ranging from 26–38 KJ/100 g. The energy levels of all assessed tea plants imply they can be categorized as low energy beverages. The indigenous tea plants with low calories may be good for health-conscious tea drinkers and other health-conscious persons. Most low-calorie beverages contain synthetic sweeteners that may not be beneficial for the intended purposes [37]. However, the indigenous tea plants, except DT-03 and DT-07 that had relatively higher fat values, are found as naturally low-calorie beverages and can be good for diabetics and weight and health-conscious consumers (Table 3B). 

Flavonoids play important biological activities in living systems [38,39]. They protect cells from stress, UV light, act as signaling molecules, detoxifying agents and antimicrobial defensive compounds [40]. The seven indigenous tea plants (Table 2) were rich in total flavonoids. The maximum TFC was found in DT-01. The richness of the indigenous tea plants in flavonoids could be beneficial to dermatology conscious person as they may keep cells younger looking. Polyphenols are the secondary plant metabolites that possess outstanding antioxidant properties, suggesting a possible protective role against free radical formation and free radical-induced damage in humans [41]. Polyphenols are beneficial compounds found in many plant foods and are grouped as flavonoids, phenolic acid, polyphenolic amides, and other polyphenols. Polyphenols are potent antioxidants and free radical scavengers [42]. In this study, FRAP, ORAC, total polyphenols, flavonols, flavanols, total caffeine, and TEAC were also estimated in all tea samples (Figure 2 and Figure 3). The highest content of total polyphenols equivalent to gallic acid was found in DT-06 as 349.138 µmol AAE/g. However, for total flavanols estimation as catechin equivalent found negligent to no flavanol content (<1 mg CE/g) and for TFC and flavonols equivalent to quercetin estimation content for DT-06 were found above the average value (Figure 3A–D). Flavonoid abundant, caffeine-free tea, and tea with high nutritional properties ensure the maximum health benefit and creates confidence in the product [36]. These tea plants can be used in beauty products due their unique phytochemical contents as a result of their strong antioxidant property.

Hypoxia is contemporary evidence for a stimulator of migration and invasion in a wide range of tumor cells in tumor microenvironments [10,11]. During carcinogenesis, low levels of oxygen may arise, boosting tumor cell proliferation, angiogenesis, and metastasis through a complex network involving HIF-1 and associated oxidative stress pathways [42]. COX-2 is a reversible enzyme required for the conversion of arachidonic acid to prostaglandins (PGs) and found abundantly in a wide range of human cancers and cancer cell lines to promote vasodilation and immune cell infiltration [43]. CoCl2-induced U87 MG cells was used in this study to check cell growth and any effect on suppression of COX. DT 1, DT-2, and DT-04 concentration dependently reduced the COX (* *p* < 0.05) as compared to CoCl2-induced U87 MG cells (Can we refer to the figure with these results).

## 4. Methodology

### 4.1. Collection of Tea Plants, Authentication, and Extraction

Seven indigenous tea plants (Coded: DT-01 to DT-07. Supplementary Figure S1, Table 2) from the Eastern Cape and North West Provinces of South Africa were studied. The plants voucher specimen was deposited at Geo Potts (BLFU) herbarium, University of the Free State (Table 2). The tea plants were prepared by infusion following the producer’s instructions and traditionally prepared by infusion in boiled water for 3–5 min. Each dried tea sample (2 g) was infused in 200 mL of boiled water for 5 min. The filtrates were freeze-dried, and their extractabilities calculated as *w*/*w*. The dried extracts were stored in a cold room (4 °C) for further experimental use. The samples were stored in cold room in sterile airtight glass containers, and for use in further experiment within couple of days and during repetition of the study, we followed the biochemical assays after storing them for 15 days and 40 days and we found no significant changes.

### 4.2. Antioxidant Screening Potential

#### 4.2.1. Ferric Reducing/Antioxidant Power Assay

Ferric reducing/antioxidant power (FRAP) assay was performed according to Benzie and Strain [44]. A reaction mixture containing 10 µL tea extract or standard (Ascorbic acid (0–1000 µM)) and 300 µL of FRAP reagent was incubated at 37 °C for 30 min. Absorbance was measured at 593 nm. The reducing power activity of the tea was interpolated from the ascorbic standard curve.

#### 4.2.2. Trolox Equivalent Antioxidant Capacity (TEAC) Assay

ABTS (2,2′-azino-di-3-ethylbenzthialozine sulphonate) radical cation scavenging assay was performed to estimate TEAC [45]. Decolorization of ABTS radical was measured at 734 nm after mixing 25 µL of tea extract or standard (Trolox, 0–500 µM) with 275 µL ABTS (absorbance of 1 at 734 nm). Antioxidant activity of tea plants was interpolated from Trolox standard curve.

#### 4.2.3. Oxygen Radical Absorbance Capacity (ORAC) Assay

The oxygen radical absorbance capacity of tea plants was determined using the method described by Ou et al. [46]. Fluorescein (48 nM. 138 µL) was mixed with 12 µL of tea plants or standard (Trolox. 0–25 µM) and 50 µL AAPH (2. 2′-Azobis (2-amidinopropane) dihydrochloride, 25 mg/mL). Decay in fluorescence was measured over 2 h with excitation wavelength set at 485 nm and emission wavelength set at 538 nm. The antioxidant activity of tea plants was extrapolated from the Trolox standard curve prepared.

### 4.3. Nutritional Content Estimation

The nutritional composition of tea plants was measured using standard methods at the Agricultural Research Council (ARC), South Africa (ARC-Irene Analytical Services. Pretoria South Africa; Facility Accreditation Number: T0063) based on South African National Accreditation System (SANAS) accredited methods. For dry matter and moisture content (ASM 013) [47], Ash (ASM 048) [34]. Fat determination using the ether extraction (ASM 044) [47]. Protein estimation determined by nitrogen content 6.25 (ASM 078) [48]. Calculated carbohydrate and energy (ASM 075) [49]. Calcium (ASM 042) [42]. Total non-structural carbohydrates (ASM 073). Others include starch [50]. Total sugars, water-soluble carbohydrates, glucose, fructose, sucrose, maltose, lactose [50]. Dietary fiber (ASM 070) [51], tryptophan (ASM 022), cysteine [52] and amino acids (arginine, serine, aspartic acid, glutamic acid, glycine, threonine, alanine, tyrosine, profile, HO-proline, methionine, valine, phenylalanine, isoleucine, leucine, histidine, and lysine) (ASM 021) [6,47].

### 4.4. Energy Determination

First, infused tea plants (2.5 g in 250 mL of boiling water) were decanted after 5 min. Energy calculations were performed using total solids (ASM 0130), moisture (ASM 0139) [47], Ash value (ASM 044) [47]. Ether Fat extraction (ASM 044) [48] and energy were calculated using (ASM 076) [34] in Kj/100 g. The energy was calculated as follows:

Energy (kJ/100 g) = 37(%Fat) + 17(%Protein) + 17(% Carbohydrates)(1)

### 4.5. Quantitative Phytochemical Determination of the Tea Plants

#### 4.5.1. Total Polyphenol Content (TPC) Estimation

Total phenolic content was estimated by previously described protocols [52]. A total of 25 μL of extract or standard reagent (Gallic acid) and 125 μL of Folin reagent (0.2 M) were mixed with 100 μL of a 7.5% solution of sodium carbonate and incubated for 2 h at RT. Absorbance was measured at 765 nm. The results are expressed as mg of gallic acid equivalents (GAE) mg dry mass^−1^.

#### 4.5.2. Flavanol Quantification via Catechin Estimation

4-(Dimethylamino)-cinnamaldehyde (DMACA) was used to quantify flavanols according to a previous method described by Li et al. [53]. A standard curve of catechin was prepared using 0–100 µM of catechin. The reaction mixture contained 50 µL of tea or standard and 250 µL of DMACA reagent (0.25 g dissolved in 50 mL 3:1 methanol: hydrochloric acid). The reaction mixture was incubated for 30 min at room temperature, and the absorbance recorded at 640 nm. Catechin present in tea plants was extrapolated from the standard curve prepared using the standard absorbance of catechin. 

#### 4.5.3. Flavonol Quantification via Quercetin Estimation

Flavonols were quantified using the method described by Mazza et al. [54]. About 250 µL reaction mixture containing 225 µL of 95% ethanol, 12.5 µL of 0.1% HCl, and 12.5 µL of tea extract or standard (0–80 mg/L Quercetin) was prepared. After 30 min incubation at room temperature, absorbance was measured at 360 nm. Quercetin present in tea plants was extrapolated from the quercetin standard curve.

### 4.6. Cytotoxicity and Cell Proliferation 

#### 4.6.1. Cell Proliferation Assay

The safety of the indigenous tea plants was assessed at the cell level using the MTT assay [55]. The extracts of the tea plants were diluted to a final concentration range of 50–2000 µg/mL and 31.25–500 µg/mL for HEK293 and Chang liver cell lines, respectively. Doxorubicin (1–100 µg/mL) and verapamil (6.25–100 µg/mL) were used as positive controls. U87 glioblastoma cells were seeded in 96-well plates at 1 × 10^6^ cells/well containing 200 µL DMEM, supplemented with 10% fetal bovine serum (FBS), 100 U/mL penicillin, and 100 µg/ ml streptomycin (Gibco, MA, USA).

After 24 h treatment period, cells were exposed to MTT reagent (0.5 mg/mL). The colorimetric reaction was measured utilizing a plate reader (Multiskan Go. Thermofischer Scientific, Vantaa, Finland) at 570 nm wavelength. The samples were evaluated in three independent experimental repeats, and each sample was assessed in triplicate. Cell proliferation was calculated from the absorbance readings. 

#### 4.6.2. Cell Counts Assay

Cells were seeded at 1 × 10^5^ in a 35-mm plate and after 6 h the cells were treated with tea plants (DT01-DT07) hot infusion extract at 250 μg/mL. The cells and medium were collected at 24 h and 48 h and stained with 0.4% trypan blue to distinguish viable from unviable cells. Cell counts were performed by cell counter (Life Technologies Countess II FL Cell Counter. Standwood. WA). Cells were counted after three times treatment.

#### 4.6.3. Cyclooxygenase (COX) Activity Assay

COX inhibition was evaluated using flurometric inhibitor screening kits (Biovision. Switzerland) following the manufacturer’s instruction. Briefly, to prepare cell lysate, cells (~2–3 × 10^5^) were washed with 10 mL PBS (1×). After centrifuging at 500× *g* for 3 min, supernatant was discarded and the suspended cell pellet was exposed to 0.5 mL of lysis buffer with protease inhibitor cocktail (Sigma-Aldrich, St. Louis, MO, USA). Cells were treated with different extracts at different concentrations (50, 100, and 250 µg/mL) after overnight exposure in CoCl_2_ at 100 µM.

### 4.7. Statistical Analysis 

Standard curve was prepared using Graph Pad Prism software version 4.03. Linear regression was performed using the effect of “force zero” and interpolation of unknowns from a standard curve with linear regression “Slope = 0.01698 ± 0.0004994” and R^2^ > 0.991. Statistical significance (*p*) was determined using one-way ANOVA followed by Dunnett’s multiple post hoc test of significance where * *p* < 0.05. Graph Pad Prism version 4.03 and Graph Pad Instat software (Graph Pad Software Inc., San Diego, CA, USA) and Origin 6 (Ameland, The Netherlands) were the statistical software used.

## 5. Conclusions

The health benefits of these tea plants could be associated with their strong antioxidant activities as well as because of their nutritional and nutraceutical properties. Moreover, the African indigenous tea plants have health beneficial role as they have shown in this research to inhibit the COX activity that could play a role in cancers. Widespread research during the last two decades has explored the mechanism of oxidative stress that can lead to chronic inflammation, which further possibly will mediate most chronic diseases including cancers. Therefore, the indigenous low calorie tea infusion may have an added benefit to health-conscious consumers due to COX inhibition and its role in and conquest the toxic autochthonous tissue free radicals. In conclusion, their study has found the tea plants to be non-cytotoxic, have antioxidant properties, and found to containing nutritional phytochemicals with low fat and calorie value. These findings point to an overall benefit in the consumption of these tea plants. There is a need for further development of the indigenous tea plants to improve their aroma and flavor. The indigenous tea plants have a reasonable market potential to compete with the current commercial brands. The University of the Free State, South Africa, is vigorously pursuing the development of the seven indigenous tea plants (Supplementary Figures S2–S8) for possible commercialization.

## Figures and Tables

**Table 1:** Selected indigenous South African indigenous tea plants with their code names, botanical names, local names, uses and geographical coordinates.

S/N (Plant Codes)	Botanical Names	Family Names	Local Names	Common Names	Uses	Geographical Coordinates	References
(DT-01)	*Lippia javanica* (Burm, f.) Spreng	Verbenaceae	iNzinziniba (Xhosa); uMsuzwane. uMswazi (Zulu). Koorsbossie, Beukesbossie, Lemoenbossie (Afrikaans)	fever tea, lemon bush	herbal health tea to treat colds, cough, fever or malaria, wounds, repelling mosquitos, diarrhoea, chest pains, bronchitis, and asthma; widely sold as herbal tea in Botswana, South Africa. and Zimbabwe; In South Africa used in the treatment of livestock ailments; Isolated (E)-2(3)-tagetenone epoxide from *L. javanica* inhibited the HIV-1 reverse transcriptase enzyme. Leaf and root decoction or infusions used to remedy digestive system diseases (cholera, diarrhoea, and dysentery). Also to repel insects	*29°05′32.2′ S; 26°09′25.6′ E*	[3,12,13,14,15]
(DT-02)	*Lippia scaberrimma* (Sond,)	Verbenaceae			In Malawi and Zimbabwe to treat coughs, asthma, fever, and headaches; in Botswana as a soothing and relaxing tea; essential oils have fungistatic activity against *Botryosphaeria parva* and *Colletotrichum gloeosporioides*	*29°05′32.2′ S; 26°09′25.6′ E*	[16]
(DT-03)	Under evaluation	Under evaluation	Iti ye Hlathi (Local name of plant in Uitenhage in EC Province of South Africa)		Locals in Uitenhage area. Eastern Cape used it as a beverage (with or without sugar and milk). THPs use the tea to treat and manage swollen legs and feet, body itches and body pains. Also used to boost energy; to clean the womb, kidneys and blood, uretic and an antihypertensive.	*32.2968° S, 26.4194° E*	Our Lab report (not published)
(DT-04)	*Buddleja saligna* (L)	Scrophulariaceae	Witolien (Afrikaans). unGqeba (Xhosa). iGqeba-elimhlope (Zulu)	False olive	To treat high blood pressure. chest pains, coughs, diabetes, colds, and tuberculosis; anti-inflammatory, anti-malaria, antioxidant, and antibacterial activities; anti-microbial acetylcholinesterase inhibitory activities; eye infections and neurodegenerative diseases; antiplasmodial activity, inhibition of nitric oxide (NO) overproduction, and anti-proliferative activity	*32°43′25.5′ S; 26°53′28.2′* E	[17,18,19,20,21]
(DT-05)	*Phyla dulcis* (Trev.)	Verbenaceae	Hawu Hawu by amaXhosa people of Ntshatshongo	bushy lippia, honey herb	As natural sweetener; to treat various diseases associated with bronchi; to manage cough and an emmanagogue; asthma and chronic bronchitis; has a minty taste and do not cause tooth decay	*29°09′18.01′ S; 26°16′25.63′ E*	[12]
(DT-06)	*Myrothamnus flabelifollius*(Welw.)	Myrothamnaceae	Uvukakwabafile (isiZulu). Bergboegoe (Afrikaans) Moritela Tshwene (Setswana)	resurrection plant	Nama people use the leaf extract in wound healing, asthma and chest ailments; Also for infectious and respiratory diseases, inflammation, heart, and kidney ailments; to treat chest pains, coughs, epilepsy, and mental disorders; As tonic and skin moisturizer; antidiabetic and antioxidant activity; potent antimicrobial activity; antioxidant, anticancer, antiviral, antidiabetic, anti-inflammatory, antiarthritic, antiulcer and antimicrobial properties	*25°31′84.1′ S; 26°11′68.3′ E*	[4,22,23,24,25]
(DT-07)	*Croton gratissimus var gratissimus (L.)*	Euphorbiaceae	Bergboegoe. iLabele. iNkubathi	Lavender croton	anti-microbial activity; sexually transmitted diseases; antiplatelet aggregation. anti-proliferative activities and antiplasmodial activities; influenza, colds, fevers; antioxidant and acetylcholinesterase inhibitory activity	*25°32′85.72′ S; 26°12′32.85′ E*	[26,27,28,29,30,31,32,33,34,35,36,37,38,39]

**Table 2:** Indigenous tea plants (Code: DT-01 to DT-07) from different regions of South Africa. Voucher specimen. Trade names and infusion extractabilities.

Indigenous Tea Plants (Infusions)					
Code	Plant Species (Local Name)	Botanical Name	Voucher Specimen No. BLFU	Trade Name	Water Extraction Yield (%)
DT-01	Inzinziniba	*Lippia javanica*	MGM005	Zinibar	7.36
DT-02	Mosukujane	*Lippia scaberrimma*	MGM0012	Mosukujane	15.48
DT-03	Iti Yehlathi	*Iti Yehlathi*	MGM008	Tinar	11.4
DT-04	Igqhange	*Buddleja saligna*	MGM0015	Gancair	7.1
DT-05	Hawu Hawu	*Phyla dulcis*	MGM0016	Haw Haw	12.22
DT-06	Moritela Tshwene	*Myrothamnus flabellifolius*	MGM0011	Resurrection bush	14.08
DT-07	Moologa	*Croton gratissimus* var *gratissimus*	MGM009	Moologa	14.74

**Table 3:** Nutritional content of all the tea plants. (**A**) indigenous tea plants. (**B**) Nutritional and nutraceutical properties of all the tea samples.

**A**									
**Analysis**	**Method Number**	**Unit**	**Tea Sample**						
			**DT-01**	**DT-07**	**DT-03**	**DT-04**	**DT-02**	**DT-05**	**DT-06**
Dry matter	ASM 013	%	89.56	92.68	91.02	94.51	91.26	91.49	93.46
Moisture	ASM 013	%	10.41	7.32	8.98	5.49	8.74	8.51	6.54
Ash	ASM 048	%	9.22	5.13	13.14	3.65	9.56	14.20	3.22
Protein (N × 6.25)	ASM 078	%	14.44	12.80	14.12	6.15	10.09	17.60	9.26
Fat (Ether extraction)	ASM 044	%	1.33	3.43	2.84	2.90	2.83	3.19	3.81
Carbohydrates (Calculated)	ASM 075	%	64.60	71.32	60.92	81.81	68.78	56.50	77.17
Energy (Calculated)	ASM 076	kJ/100 g	448	583	462	529	586	506	771
Calcium	ASM 042	%	1.49	0.89	2.13	1.06	1.29	1.69	0.59
Total Non-structural Carbohydrates	ASM 074	%	9.03	14.03	6.85	18.67	18.25	5.21	27.81
Water Soluble Carbohydrates	NSA	%	0.04	0.05	0.03	0.10	0.10	0.02	0.16
Starch	NSA	%	0.00	0.00	0.00	0.00	1.17	0.00	0.00
Total sugars	NSA	%	0.03	14.03	6.85	18.67	17.08	5.21	27.81
Glucose	NSA	g/100 g	0.00	0.00	0.00	0.00	2.71	0.00	4.59
Fructose	NSA	g/100 g	0.00	0.00	0.00	0.00	2.93	0.00	2.94
Sucrose	NSA	g/100 g	0.00	0.00	0.00	2.53	2.77	0.00	0.00
Maltose	NSA	g/100 g	0.00	0.00	0.00	0.00	0.00	0.00	0.00
Lactose	NSA	g/100 g	0.00	0.00	0.00	0.00	0.00	0.00	2.84
Dietary Fibre (Total)	ASM 070	%	61.86	55.09	43.29	50.29	46.40	48.77	33.40
Cysteine	NSA	g/100 g	0.22	0.26	0.24	0.16	0.19	0.34	0.21
Tryptophan	ASM 022	g/100 g	0.06	0.05	0.06	0.04	0.04	0.10	0.07
Arginine	ASM 021	g/100 g	0.74	0.65	0.83	0.35	0.56	1.01	0.58
Serine	ASM 021	g/100 g	0.56	0.45	0.65	0.32	0.49	0.90	0.45
Aspartic acid	ASM 021	g/100 g	1.25	0.87	1.32	0.54	0.83	1.68	0.80
Glutamic acid	ASM 021	g/100 g	1.34	1.20	1.35	0.58	0.99	1.82	0.96
Glycine	ASM 021	g/100 g	0.62	0.47	0.71	0.33	0.50	0.96	0.48
Threonine	ASM 021	g/100 g	0.53	0.41	0.60	0.27	0.42	0.80	0.41
Alanine	ASM 021	g/100 g	0.66	0.50	0.73	0.32	0.51	0.96	0.50
Tyrosine	ASM 021	g/100 g	0.49	0.36	0.53	0.24	0.40	0.64	0.34
Proline	ASM 021	g/100 g	0.75	0.69	0.64	0.48	1.26	0.87	0.45
HO-Proline	ASM 021	g/100 g	0.10	0.13	0.16	0.07	0.08	0.20	0.10
Methionine	ASM 021	g/100 g	0.18	0.13	0.18	0.08	0.14	0.21	0.14
Valine	ASM 021	g/100 g	0.70	0.51	0.76	0.34	0.53	0.96	0.49
Phenylalanine	ASM 021	g/100 g	0.66	0.54	0.73	0.31	0.48	0.94	0.43
Isoleucine	ASM 021	g/100 g	0.58	0.43	0.61	0.29	0.44	0.78	0.41
Leucine	ASM 021	g/100 g	0.98	0.74	1.10	0.49	0.75	1.40	0.73
Histidine	ASM 021	g/100 g	0.76	0.46	0.50	0.35	0.57	0.78	0.29
Lysine	ASM 021	g/100 g	0.76	0.66	0.62	0.35	0.68	0.85	0.57
**B**									
**Sample**	**Sample Names**		**Total Solid (%)**	**Moisture (%)**	**Ash Value (%)**	**Fat (%)**	**Energy KJ/100 g**		
DT-01	*Lippia javanica* (Burm.f.)		0.25	99.75	0.01	0.78	29		
DT-02	*Lippia scaberrimma* (Sond.)		0.19	99.81	0.01	1.03	38		
DT-03	*Iti Yehlathi*		0.35	99.65	0.01	0.79	29		
DT-04	*Buddleja saligna* (L.)		0.26	99.74	0.01	1.01	37		
DT-05	*Phyla dulcis* (Trevir.)		0.11	99.89	0.01	1.06	39		
DT-06	*Myrothamnus flabellifolius* (Welw.)		0.38	99.64	0.01	0.95	35		
DT-07	*Croton gratissimus var gratissimus* (L.)		0.25	99.75	0.01	0.71	26		

## Data Availability

Not Applicable.

## Supplementary Materials

The following supporting information can be downloaded at: https://www.mdpi.com/article/10.3390/molecules27113505/s1, Figure S1: (a. DT-03) *Iti ye Hlathi*. (b. DT-05) *Phyla dulcis* (Trev.). (c. DT-01) *Lippia javanica *(Burm. f.) Scheme 04. *Buddleja saligna* (L). (e. DT-07) *Croton gratissimus var gratissimus* (L.). (f. DT-02) *Lippia scaberrimma* (Sond.). (g. DT-06) *Myrothamnus flabelifollius* (Welw.), Figure S2: DT-01 (*Lippia Javanica*; Zanibar): A: Tea branding & B: Infusion branding of the Product; of having 20 tea bags of 2.5 g each, Figure S3: DT-02 (*Lippia Scaberrimma*; Mosukujane): A: Tea branding & B: Infusion branding of the Product; of having 20 tea bags of 2.5 g each, Figure S4: DT-03 (*Iti Yehlathi*; Tinar): A: Tea branding & B: Infusion branding of the Product; of having 20 tea bags of 2.5 g each, Figure S5: DT-04 (*Buddleja saligna*; Gancair): A: Tea branding & B: Infusion branding of the Product; of having 20 tea bags of 2.5 g each, Figure S6: DT-05 (*Phyla dulcis*; Haw Haw): A: Tea branding & B: Infusion branding of the Product; of having 20 tea bags of 2.5 g each, Figure S7: DT-06 (*Myrothamnus flabellifolius*; Resurrection bush): A: Tea branding & B: Infusion branding of the Product; of having 20 tea bags of 2.5 g each, Figure S8: DT-07 (*Croton gratissimus var gratissimus*; Moologa): A: Tea branding & B: Infusion branding of the Product; of having 20 tea bags of 2.5 g each.
