# Supplementary material for: Study on South African Indigenous Teas—Antioxidant Potential, Nutritional Content, and Hypoxia-Induced Cyclooxygenase Inhibition on U87 MG Cell Line
Source: Molecules. 2022 May 30;27(11):3505. doi: 10.3390/molecules27113505 (PMC9181930; doi:10.3390/molecules27113505)
Supplement: Supplementary file 1 [file molecules-27-03505-s001.zip › molecules-1732264-supplementary.pdf]

Supplementary figures

Study on South African Indigenous Teas—Antioxidant Potential, Nutritional Content, and Hypoxia-Induced Cyclooxygenase Inhibition on U87 MG Cell Line

Motlalepula Gilbert Matsabisa [1,*], Asis Bala [1,2], Satyajit Tripathy [1], Michelle Mogomane Digashu [1], Fanie Rautenbach [3], Barsha Dassarma [1], Joseph Omorogiuwa Erhabor [1], Fernao Castro Braga [4], Pulok Kumar Mukherjee [5], Minke Tang [6] and Youngmin Kang [7]

1 Department of Pharmacology, Faculty of Health Sciences, School of Clinical Medicines, University of Free State, 9300, Bloemfontein, South Africa; asisbala_ju@yahoo.co.in (A.B.); tripathys@ufs.ac.za (S.T.); digashumm@ufs.ac.za (M.M.D.); dassarmab@ufs.ac.za (B.D.); erhaborjo@ufs.ac.za (J.O.E.)
2 Division of Pharmacology, Guru Nanak Institute of Pharmaceutical Science and Technology, Panihati, Kolkata 700114, India
3 Faculty of Health and Wellness Sciences, Oxidative Research Institute, Cape Peninsula University of Technology, 7530, Cape Town, South Africa; rautenbachf@cput.ac.za
4 Faculty of Pharmacy, Universidade Federal de Minas Gerais, 31270-901, Belo Horizonte, Brazil; fernao@netuno.lcc.ufmg.br
5 School of Natural Product Studies, Department of Pharmaceutical Technology, Jadavpur University, Kolkata, 700032, India; pulokm@yahoo.com
6 Department of Pharmacology, School of Pharmacy, Beijing University of Chinese Medicines, 100191, Beijing, China; tangmk@bucm.edu.cn
7 Propagation and Production of Traditional Herbal Medicines, Herbal Medicines Resources Centre, Korean Institute of Oriental Medicine, University of Science and Technology, Daejeon, 34054, Korea; youngmiin.k@gmail.com
* Correspondence: matsabisamg@ufs.ac.za; Tel.: +27-(0)51-401-7452

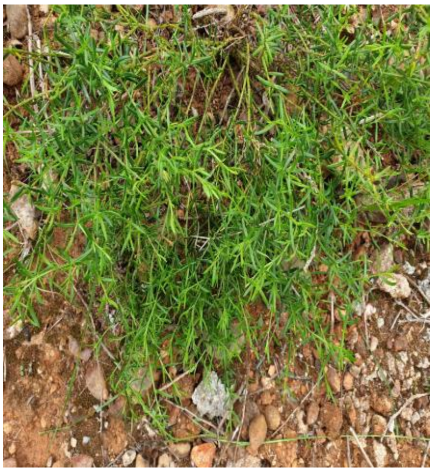
(a)

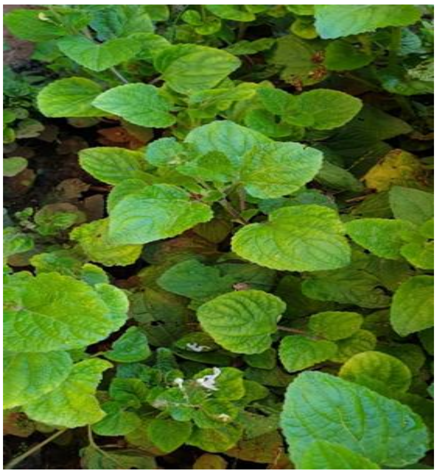
(b)

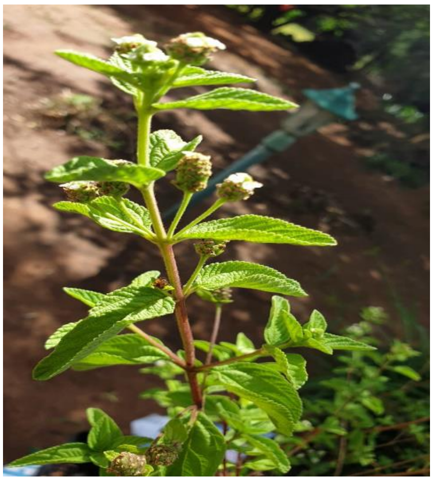

(c)

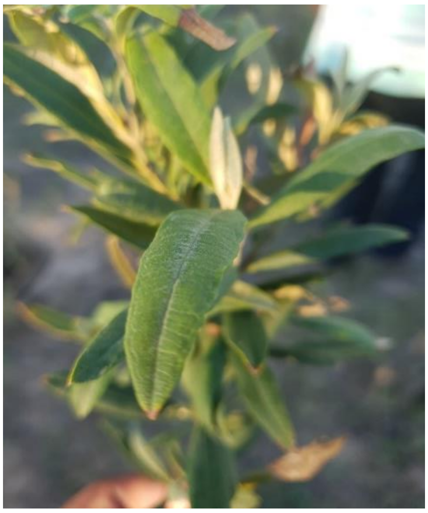

(d)

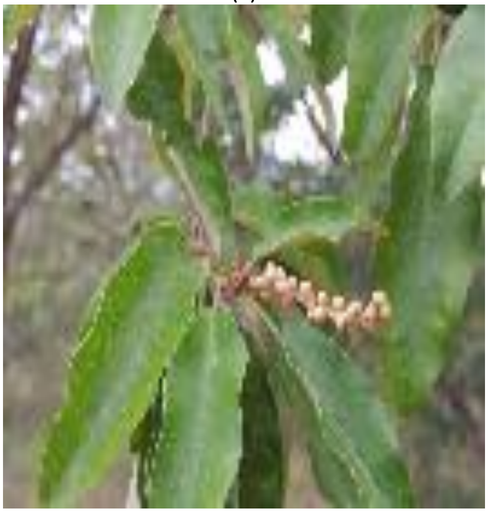

(e)

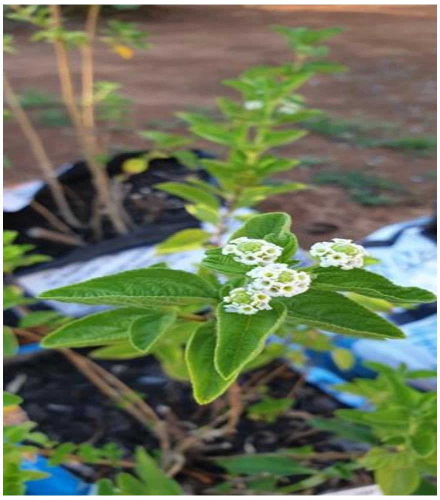

(f)

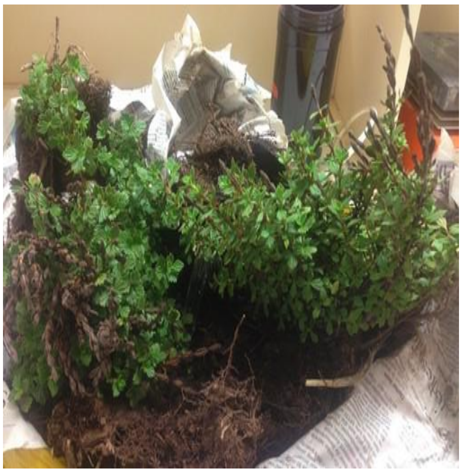

(g)

Figure S1. (**a**. DT-03) Iti ye Hlathi. (**b**. DT-05) Phyla dulcis (Trev.). (**c**. DT-01) Lippia javanica (Burm. **d**.) Scheme 04. Buddleja saligna (L). (**e**. DT-07) Croton gratissimus var gratissimus (L.). (**f**. DT-02) Lippia scaberrimma (Sond.). (**g**. DT-06) Myrothamnus flabelifollius (Welw.).

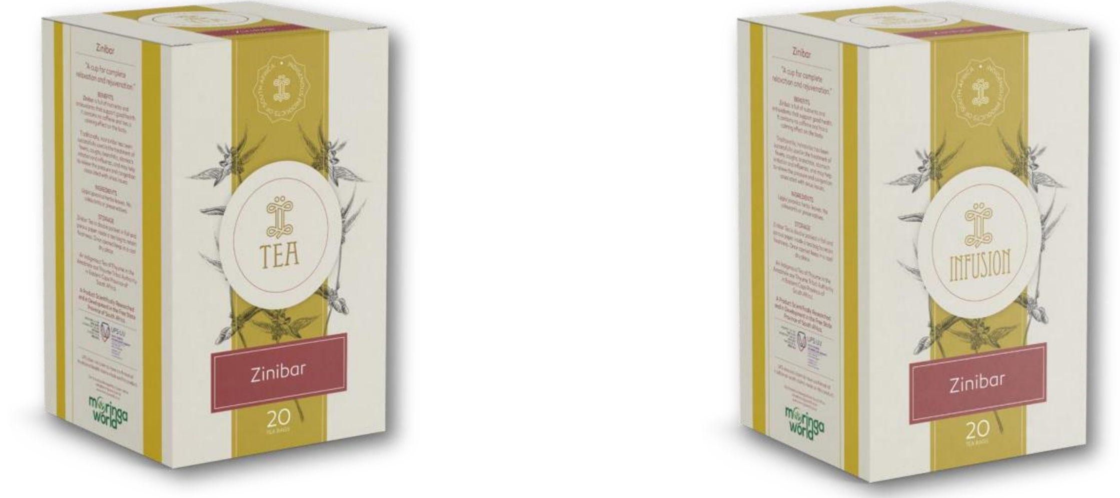

A B

Figure S2. DT-01 (*Lippia Javanica;* Zanibar): **A**: Tea branding & **B**: Infusion branding of the Product; of having 20 tea bags of 2.5g each.

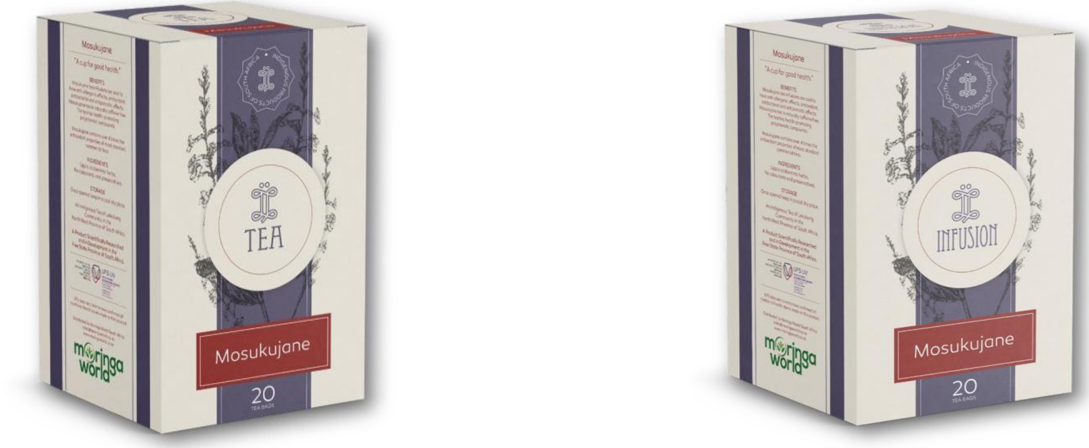

A B

Figure S3. DT-02 (*Lippia Scaberrimma;* Mosukujane): **A**: Tea branding & **B**: Infusion branding of the Product; of having 20 tea bags of 2.5g each.

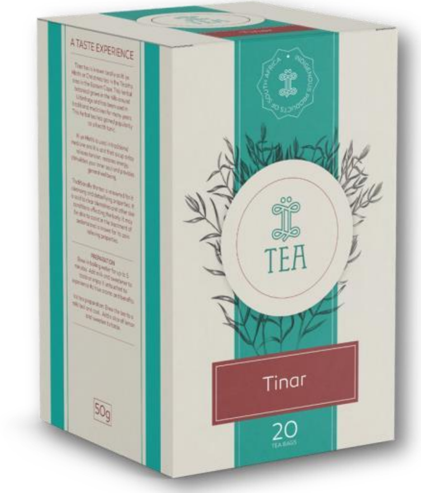

A

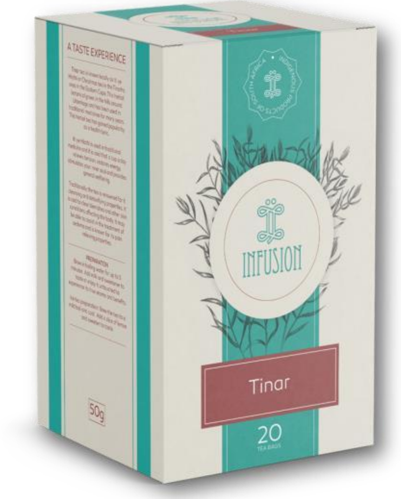

B

Figure S4. DT-03 (*Iti Yehlathi;* Tinar): **A**: Tea branding & **B**: Infusion branding of the Product; of having 20 tea bags of 2.5g each.

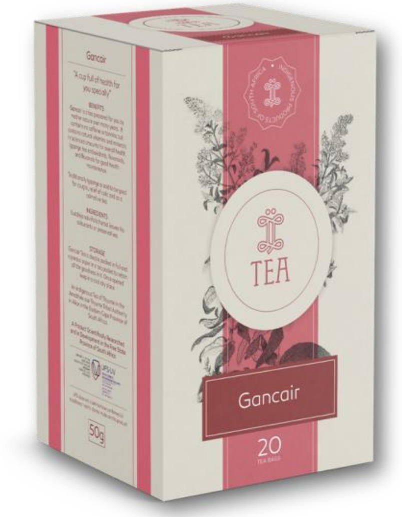

A

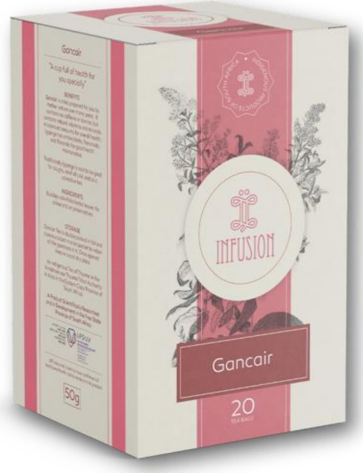

B

Figure S5. DT-04 (*Buddleja saligna;* Gancair): **A**: Tea branding & **B**: Infusion branding of the Product; of having 20 tea bags of 2.5g each.

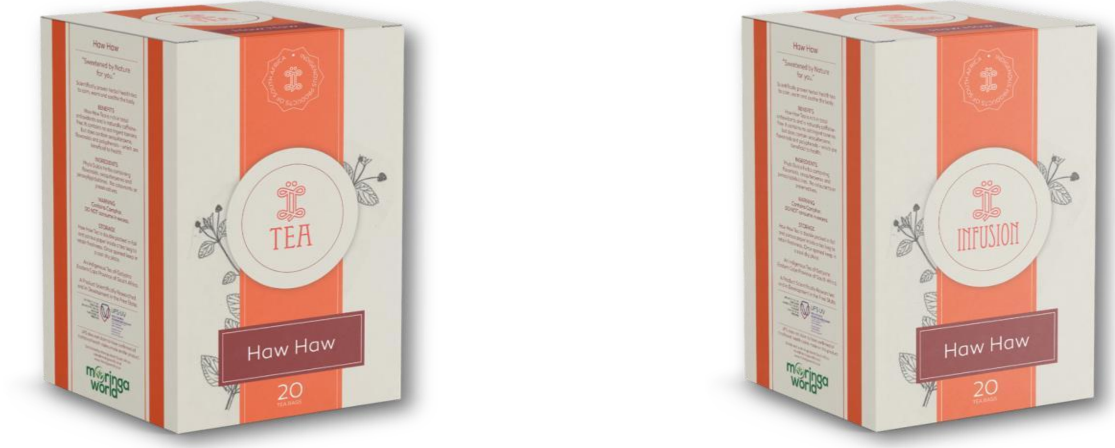

A B

Figure S6. DT-05 (*Phyla dulcis;* Haw Haw): **A**: Tea branding & **B**: Infusion branding of the Product; of having 20 tea bags of 2.5g each.

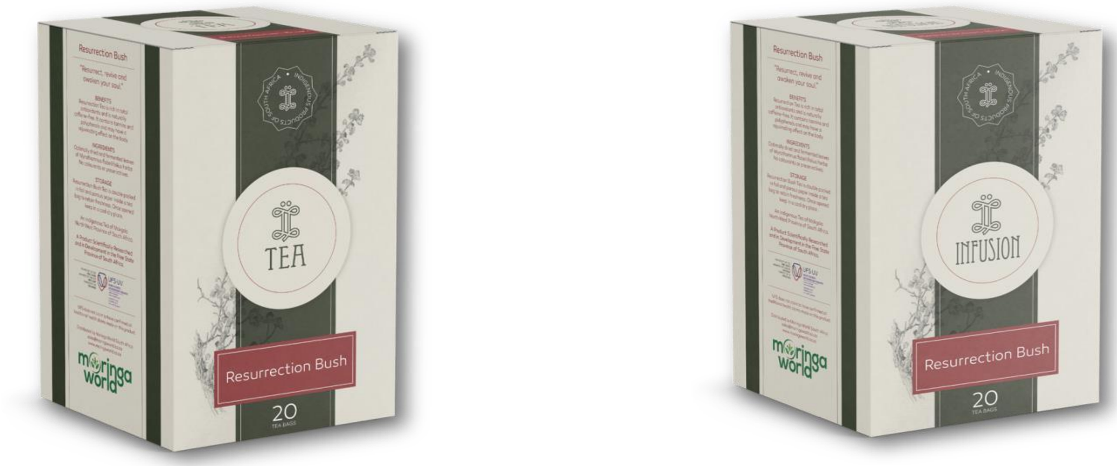

A B

Figure 7. DT-06 (*Myrothamnus flabellifolius;* Resurrection bush): **A**: Tea branding & **B**: Infusion branding of the Product; of having 20 tea bags of 2.5g each.

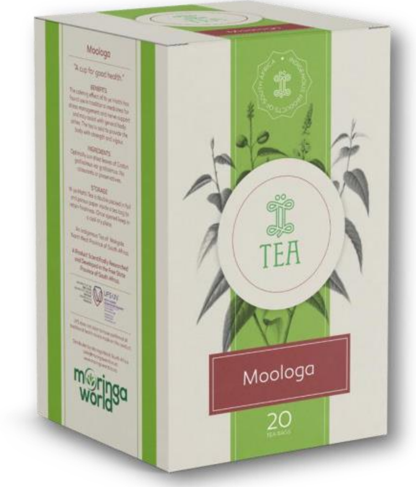

A

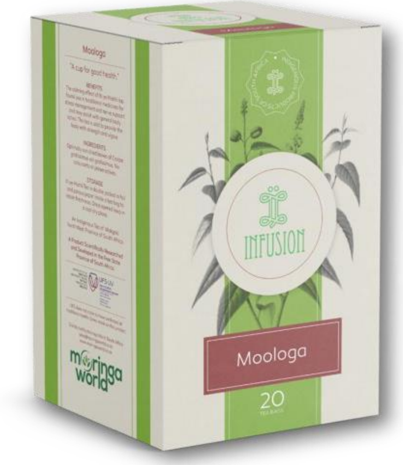

B

Figure S8. DT-07 (*Croton gratissimus var gratissimus;* Moologa): **A**: Tea branding & **B**: Infusion branding of the Product; of having 20 tea bags of 2.5g each.
